# Do Low-Mercury Terrestrial Resources Subsidize Low-Mercury Growth of Stream Fish? Differences between Species along a Productivity Gradient

**DOI:** 10.1371/journal.pone.0049582

**Published:** 2012-11-16

**Authors:** Darren M. Ward, Keith H. Nislow, Carol L. Folt

**Affiliations:** 1 Department of Biological Sciences, Dartmouth College, Hanover, New Hampshire, United States of America; 2 Center for Research on Ecosystem Change, U.S. Department of Agriculture-U.S. Forest Service, Amherst, Massachusetts, United States of America; Université de Sherbrooke, Canada

## Abstract

Low productivity in aquatic ecosystems is associated with reduced individual growth of fish and increased concentrations of methylmercury (MeHg) in fish and their prey. However, many stream-dwelling fish species can use terrestrially-derived food resources, potentially subsidizing growth at low-productivity sites, and, because terrestrial resources have lower MeHg concentrations than aquatic resources, preventing an increase in diet-borne MeHg accumulation. We used a large-scale field study to evaluate relationships among terrestrial subsidy use, growth, and MeHg concentrations in two stream-dwelling fish species across an in-stream productivity gradient. We sampled young-of-the-year brook trout (*Salvelinus fontinalis*) and Atlantic salmon (*Salmo salar*), potential competitors with similar foraging habits, from 20 study sites in streams in New Hampshire and Massachusetts that encompassed a wide range of aquatic prey biomass. Stable isotope analysis showed that brook trout used more terrestrial resources than Atlantic salmon. Over their first growing season, Atlantic salmon tended to grow larger than brook trout at sites with high aquatic prey biomass, but brook grew two-fold larger than Atlantic salmon at sites with low aquatic prey biomass. The MeHg concentrations of brook trout and Atlantic salmon were similar at sites with high aquatic prey biomass and the MeHg concentrations of both species increased at sites with low prey biomass and high MeHg in aquatic prey. However, brook trout had three-fold lower MeHg concentrations than Atlantic salmon at low-productivity, high-MeHg sites. These results suggest that differential use of terrestrial resource subsidies reversed the growth asymmetry between potential competitors across a productivity gradient and, for one species, moderated the effect of low in-stream productivity on MeHg accumulation.

## Introduction

Stream food webs are tightly linked to terrestrial food webs in nearby riparian zones through the exchange of resource subsidies [1]. For example, many stream-dwelling salmon and trout species (Family Salmonidae, or salmonids) eat terrestrial insects that fall into streams, while terrestrial spiders and birds in riparian areas eat adult aquatic insects that emerge to breed [2]. Such subsidies are often energetically important for consumers, but they may also be a key pathway for the transfer of toxic contaminants [3]. Methylmercury (MeHg) and other contaminants that accumulate predominantly in aquatic food webs are transferred into terrestrial food webs when terrestrial predators eat aquatic prey [4]. This contaminant flux out of aquatic systems has raised substantial concern as a health risk for terrestrial organisms [5]. Yet, resource subsidies go both directions, and very few studies have addressed the effect of the reciprocal subsidy of terrestrial prey into aquatic environments on accumulation of MeHg and other contaminants in fish [6].

Previous studies have identified situations in which subsidies are important food resources for particular species or communities [2], [7]. For example, terrestrial subsidies may support a larger proportion of the growth of stream fish when in-stream productivity is limited ([8], but see [7]). However, this generality does not apply to all of the consumers in the aquatic community. While some stream salmonid species switch to terrestrial prey at times or locations where aquatic prey is rare, or vice versa, diet flexibility varies across species and other salmonids are relatively specialized on only terrestrial or only aquatic prey [9], [10]. Thus, varying in-stream productivity may drive divergence in performance among stream salmonids, as species that use terrestrial subsidies gain a substantial energetic benefit relative to aquatic prey specialists at sites with low in-stream productivity, but not at more productive sites.

Variation in in-stream productivity may also mediate the effect of terrestrial subsidies on the concentration of food-borne contaminants, such as MeHg, that accumulate in aquatic food webs. Concentrations of MeHg in aquatic organisms are often elevated at resource-poor sites with low primary production rates for at least two reasons: First, at low-productivity sites MeHg is concentrated in smaller algal biomass at the base of the food web [11], [12], leading to elevated MeHg concentrations in primary consumers and their fish predators [13], [14]. Second, reduced prey quality or increased energetic costs for consumers that specialize on aquatic resources at resource-poor sites can increase the trophic transfer rate of MeHg (ratio of MeHg concentration in a consumer and its food) [15], [16], [17]. The concentration of MeHg in terrestrial resource inputs into streams is almost universally lower than comparable aquatic resources [6], [18]. Terrestrial resource MeHg concentrations are unlikely to respond to productivity in nearby aquatic ecosystems, so the difference between the MeHg concentrations in aquatic and terrestrial resources will be magnified at sites with low in-stream productivity where MeHg is elevated. For stream fish, this suggests that those species that switch to terrestrial resources at low-productivity sites may show a substantial reduction in MeHg concentration relative to the rest of the stream food web.

We conducted a comparative field study to evaluate growth and MeHg concentrations of two stream-dwelling salmonids, brook trout (*Salvelinus fontinalis*) and Atlantic salmon (*Salmo salar*), across a gradient of in-stream productivity. Juvenile brook trout and Atlantic salmon are behaviorally and physiologically similar; both emerge as free-living fry in spring, establish and defend territories, and feed on drifting invertebrates. Previous studies suggest that juvenile brook trout and Atlantic salmon may compete for growth resources [19]. However, juvenile Atlantic salmon generally prefer aquatic prey [20] whereas brook trout tend to have a more flexible diet [21], utilizing terrestrial resources to a greater degree than Atlantic salmon [9], [22] but see [8]. We used this study system to test whether brook trout consistently use more terrestrial subsidies than co-occurring Atlantic salmon and whether these diet differences reduce the effects of low in-stream productivity on brook trout growth and MeHg concentration.

## Methods

### Ethics Statement

Fish collection and handling procedures for this study were reviewed and approved by the Dartmouth College Animal Care and Use Program under protocol 06-02-12. Care was taken to minimize pain and distress by minimizing handling time, applying anesthesia during handling, and promptly euthanizing fish collected for tissue samples. Fish sampling was authorized by permits from New Department of Hampshire Fish and Game (permit number F2007-1) and Massachusetts Department of Fish and Game. Study sites included sites on both public and private land. Access to study sites was arranged with individual landowners or agencies.

### Field Methods

In 2008, we sampled 20 sites located on 10 small (<7 m average summer width, draining <30 km^2^) stony-bottomed streams in the Connecticut River basin in the vicinity of Hanover, New Hampshire and Amherst, Massachusetts (2 sites per stream, all sites separated by >1 km). All of the streams were in predominantly forested watersheds. The focal organisms for our sampling, young-of-the-year (YOY) Atlantic salmon and brook trout, were abundant at all study sites. The YOY Atlantic salmon came from newly-hatched fry we stocked in the study streams on 6-8 May 2008 as part of an ongoing salmon reintroduction program in the study area [23]. The fry were produced at the White River National Fish Hatchery from broodstock that were the progeny of sea-run parents [24]. Atlantic salmon fry stocking was timed to approximate their switch from yolk resources to external feeding; the YOY salmon are not fed in the hatchery prior to release. Brook trout reproduce naturally in the study streams; YOY brook trout emerge from the gravel and commence feeding from late March to early June, depending on stream temperature. Adult, catchable-sized brook trout are stocked in some of the study streams, but these were not included in our sampling of YOY. Besides Atlantic salmon and brook trout, fish communities in the study streams consisted largely of minnows (Family Cyprinidae; mostly blacknose dace *Rhinichthys atratulus,* 18 sites) and slimy sculpin (Family Cottidae, *Cottus cognatus*, 13 sites). Ten other fish species were encountered infrequently (each at <8 sites). In earlier work at these sites, we have documented a large gradient in biomass of aquatic insect prey for salmonids, with sites heavily shaded by riparian forest canopy and with low alkalinity stream water having prey biomass ca. ten-fold lower than sites with an open canopy and higher alkalinity [25], [26].

We measured the population density of all fish species at each site in August 2008. Fish sampling was conducted with a Smith-Root BP-12 electrofisher at 300–500 V DC. We fished three 30-m sample reaches at each study site. Each reach was isolated with block nets at the upstream and downstream end and fished for 3–4 passes of removal sampling. All salmonids and a subset of all other species were measured to the nearest mm (total length). We used a maximum weighted likelihood technique to estimate total abundance of each species in each plot from removal data (Carle & Strub 1978). We separated YOY salmonids from older fish based on stream-specific length distributions and estimated density separately for these age classes.

Biological samples collected for mercury analysis included YOY brook trout, YOY Atlantic salmon, and aquatic and terrestrially-derived prey items. We collected five individual YOY trout and salmon from each site for mercury analysis on 15–24 September 2008 so that samples captured one full growing season of growth and mercury accumulation. For aquatic prey items, we collected three replicate samples at each site using an electrobugging technique [27] in riffle habitat (<20 cm deep, >20 cm/s surface water velocity). Each replicate aquatic prey sample consisted of the pooled catch from three locations treated with a 10 second sweep with the electrofisher anode (300–500 V DC); stunned insects drifted into a 500 um mesh Surber net held downstream. This technique yielded sufficient biomass of mayfly nymphs (mostly Baetidae and Heptageniidae) for mercury analysis (>1 mg dry mass) with little detritus. Our previous work has shown that mercury concentrations in mayflies are representative of other abundant aquatic prey items for YOY salmonids at our study sites and are closely correlated to mercury accumulation in salmon [26]. Further, baetid and heptageniid mayflies represented the bulk of the spring and early summer diet for YOY Atlantic salmon and brook trout in earlier studies at these sites ( [25], Ward, D.M. unpublished data). We also used the mean total biomass of mayflies captured in standardized electrobugging samples as an index of aquatic prey biomass available at the study sites [25], [28]. Terrestrially-derived prey, defined as those that consume primarily terrestrial vegetation, was collected at a subset of sites spanning the north-to-south spatial range of the study area. Terrestrially-derived prey included caterpillars (Order Lepidoptera) that were hand-picked from riparian vegetation (three sites) and aquatic stoneflies (Family Pteronarcyidae) that rely predominantly on terrestrial leaf litter and had a terrestrial isotope signature (three sites). All biological samples were stored on ice in acid-cleaned vials for transport. Fish were frozen immediately on return from the field. Invertebrates were sorted from detritus within 24 hours and then frozen.

Fish and prey samples were freeze dried and homogenized prior to mercury analysis. For fish samples, 0.1 g subsamples were digested in ultra-clean nitric acid in sealed, acid-cleaned Teflon vessels in a microwave reaction accelerator. Total mercury concentrations in the digested solution were measured by inductively coupled plasma mass spectrometry (ICPMS, Agilent 7500cx). Most (>95%) of the mercury in YOY trout and salmon at our sites is MeHg (D.M. Ward, unpublished data), so total mercury measurements are representative of MeHg concentrations. The proportion of total mercury that is MeHg in insects is lower and more variable than that in fish, so prey samples were measured for MeHg using isotope dilution gas chromatography-ICPMS. Quality control was ensured by analysis of certified reference materials (NIST 2976, mussel tissue, certified Hg concentration 61 ppb; mean concentration in 11 samples: 68 ppg, SE: 2.6), duplicate samples (average relative percent difference of 11 duplicates: 3.5%, SE: 0.7%), and digestion blanks with every processing batch of 20 samples. Mercury sample analyses were conducted at the Dartmouth College Trace Element Analysis facility.

For isotope analysis, a small (ca. 1 mg) subsample of the freeze-dried material from each fish and prey sample was packaged into a tin capsule and analyzed for stable carbon and nitrogen isotopes (Delta+XL isotope ratio mass spectrometer). Quality control was ensured by the analysis of standards (mesquite, USGS25, and in-house fish tissue standard material). We calculated the isotope ratios using standard delta units, relative to Peedee Belemnite (carbon) or air (nitrogen). Standard measurements averaged within 0.1 delta units of nominal values (nitrogen SE: 0.09; carbon SE: 0.05) and the average of three in-house standard measurements varied less than 0.2 delta units for nitrogen and 0.3 delta units for carbon between runs. Isotope sample analyses were conducted at the Dartmouth College Stable Isotope Geochemistry Laboratory.

Our approach of using specific prey items representative of aquatic resources (mayflies) or terrestrial resources (caterpillars, pteronarcyid stoneflies) as endpoints is indicative of the relative use of aquatic and terrestrial sources by brook trout and salmon, but does not give a quantitative picture of the contribution of aquatic and terrestrial productivity to production. The mayflies we measured as representative of aquatic prey likely consume some terrestrial organic matter [29], while pteronarcyid stoneflies likely consume some in-stream production. In general, terrestrial resource subsidies can reach insectivorous fish through at least two pathways: via terrestrial insects that fall into the stream or via terrestrial plant detritus that falls in to the stream and is eaten by aquatic insects [1], [30]. Our study does not distinguish these pathways.

### Data Analysis

Data analysis had three components: isotope data analysis to determine if brook trout ate more terrestrial prey than Atlantic salmon, growth analysis to determine if brook trout were less susceptible to suppressed growth than Atlantic salmon at low prey biomass sites, and mercury data analysis to determine if brook trout had lower MeHg concentrations than salmon at low prey biomass sites. Mercury concentration, fish size, prey biomass, and population density were log-transformed in all analyses to linearize relationships and equalize variance. Data analysis was conducted using JMP Version 9.0 (SAS Institute Inc., Cary, NC, 1989–2012).

We tested whether brook trout ate more terrestrial prey than Atlantic salmon by examining the relationships between the observed carbon and nitrogen isotope ratios of the fish and the isotope ratios predicted for the fish based on a diet composed entirely of aquatic or terrestrial prey (for carbon) or just aquatic prey (for nitrogen) at each site (see [31] for a similar approach). Predicted isotope concentrations for each diet were calculated assuming no fractionation between fish and prey for ^13^C and fractionation of 3.4‰ for ^15^N [32]. Our measurements of δ^13^C in terrestrial prey did not vary substantially across the subset of sites for which we had data, consistent with a relatively uniform δ^13^C signature in forest vegetation [33], [34], [35], so we assumed that the carbon isotope ratio in terrestrial prey was uniform at the mean of observed values (26‰) across all sites. Terrestrial δ^15^N measurements differed across the subset of sites where we collected terrestrial samples, so δ^15^N was not included in the analysis for terrestrial prey.

We tested the effects of reduced aquatic prey biomass on growth of each species using mass of fish at each site in September as an index of summer growth. This growth index encompasses total mass gain over the first growing season and is directly proportional to growth rate for stocked YOY Atlantic salmon that are all the same age. In contrast, brook trout may vary in hatching and emergence time across sites such that variation in size reflects variation in age as well as variation in growth rate. Nonetheless, size at the end of summer is still an ecologically meaningful measurement [36]. For the analysis of summer growth, we first used a separate multiple regression for each species with mean mass of YOY in September at each site as the response and prey biomass, Atlantic salmon population density, and brook trout population density as predictors. Population density of each species was included in the models to account for potential effects of density-dependent growth [25] and interspecific competition for growth resources. We also directly evaluated the difference in brook trout and Atlantic salmon growth across the prey biomass gradient by regressing the difference in mean log-transformed brook trout and salmon mass at each site against prey biomass.

For analysis of MeHg concentration, we first confirmed that terrestrial prey had lower MeHg concentrations than aquatic prey using a t-test and confirmed that MeHg concentrations in aquatic prey were higher at sites with low prey biomass using linear regression. Then, we tested whether brook trout and Atlantic salmon MeHg concentrations showed different patterns across sites by comparing the linear regressions describing the relationship between mean MeHg concentration in each fish species and MeHg concentration in aquatic prey at each site. We also directly evaluated the difference in brook trout and Atlantic salmon MeHg concentration across the prey biomass gradient by regressing the difference in mean log-transformed brook trout and salmon MeHg concentrations at each site against prey biomass.

## Results

**Figure 1:**
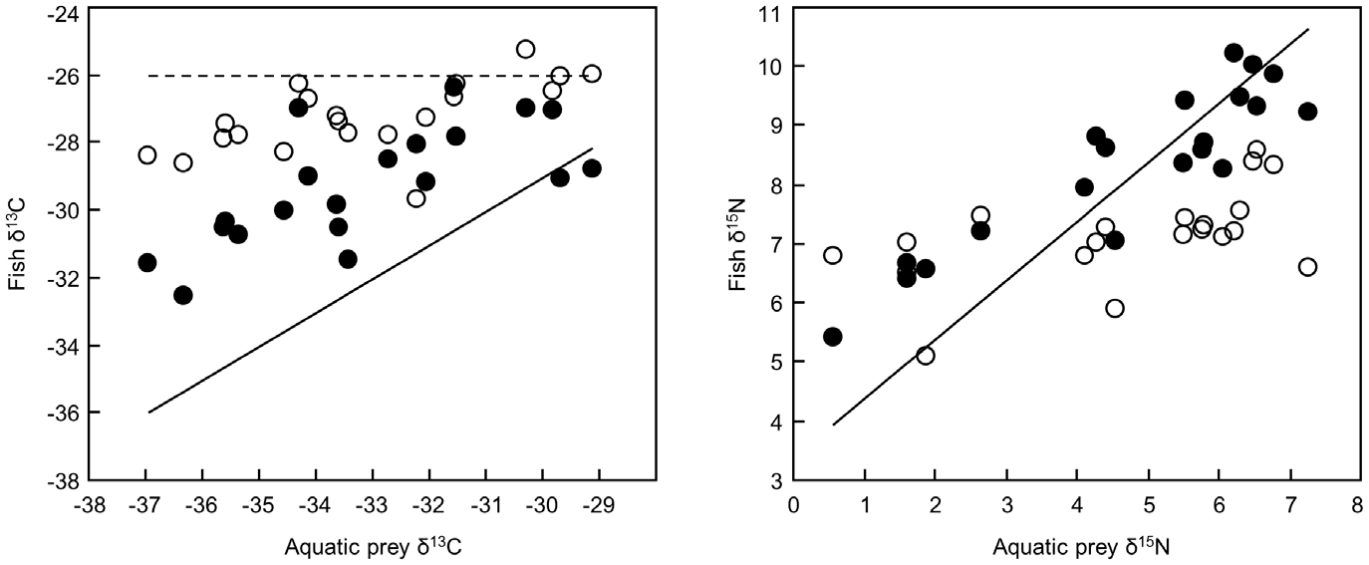
Stable isotopes indicate that brook trout rely on terrestrial subsidies more than Atlantic salmon do. Points on the scatterplots show the average carbon and nitrogen stable isotopes in brook trout (open circles) and Atlantic salmon (closed circles) at each site against carbon and nitrogen stable isotopes in aquatic prey (same prey value for both species at each site). Error bars are omitted for clarity; SEs of all estimates were less than 0.8 delta units for carbon and 0.6 delta units for nitrogen. The solid lines show the predicted stable isotopes for brook trout or Atlantic salmon with a diet consisting entirely of aquatic prey. The dashed line for carbon shows the predicted isotopes for brook trout or Atlantic salmon with a diet consisting entirely of terrestrially-derived prey.

Isotope analysis indicated that brook trout used more terrestrial prey resources than Atlantic salmon. Both brook trout and Atlantic salmon mean δ^13^C values were generally intermediate to those predicted for a fully-terrestrial or fully-aquatic diet (Figure 1). However, mean brook trout δ^13^C values were closer to predictions for a terrestrial diet and mean Atlantic salmon δ^13^C values were closer to predictions for an aquatic diet. The linear regression relationship between aquatic prey δ^13^C and brook trout δ^13^C (brook trout δ^13^C = −17.6+0.29 (aquatic prey δ^13^C), *r* ^2^ = 0.39, root mean square error (RMSE) = 0.85, *F*_1,18_ = 11.7, *P* = 0.003) was weaker and had a slope further from 1 than that for Atlantic salmon (Atlantic salmon δ^13^C = −12.0+0.52 (aquatic prey δ^13^C), *r* ^2^ = 0.49, RMSE = 1.26, *F*_1,18_ = 17.1, *P* = 0.0006), consistent with reduced reliance on aquatic resources. The intercepts of the δ^13^C regressions were significantly different between brook trout and salmon (*t* = 5.9, df = 36, *P*<0.001), but the slopes were not (*t* = 1.5, df = 36, *P* = 0.14). Similarly, brook trout δ^15^N did not correspond as closely with predicted δ^15^N for a fully aquatic diet as Atlantic salmon (Figure 1), and the regression relationship between brook trout δ^15^N and aquatic prey δ^15^N (brook trout δ^15^N = 6.14+0.22 (aquatic prey δ^15^N), *r* ^2^ = 0.29, RMSE = 0.69, *F*_1,18_ = 7.5, *P* = 0.01) was weaker than that for salmon (Atlantic salmon δ^15^N = 5.5+0.61 (aquatic prey δ^15^N), *r* ^2^ = 0.83, RMSE = 0.57, *F*_1,18_ = 90.1, *P*<0.0001). Both the intercepts (*t* = 5.9, df = 36, *P*<0.001) and slopes (*t* = 3.9, df = 36, *P*<0.001) of the δ^15^N regressions were significantly different between brook trout and salmon.

**Figure 2:**
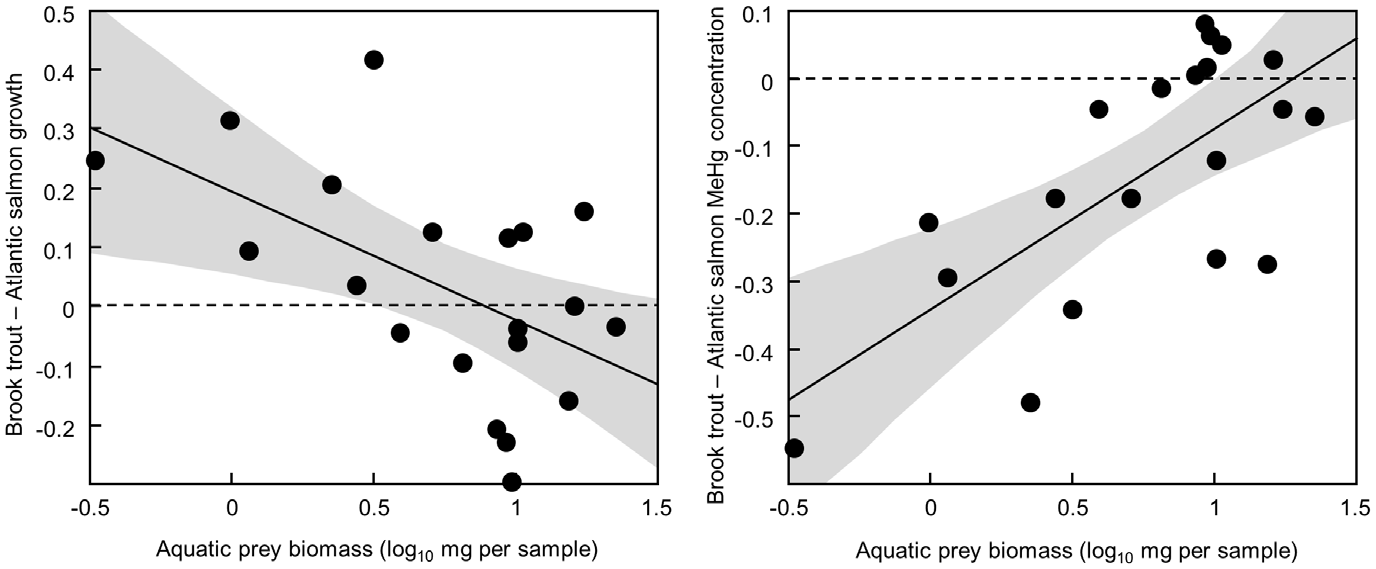
Brook trout and Atlantic salmon respond differently to reduced aquatic prey biomass. Points on the scatterplots show the difference between brook trout and Atlantic salmon growth or methylmercury (MeHg) concentration at each site against aquatic prey biomass. Each point represents the difference between the mean values for the two species at one of the study sites. Positive values indicate that means for brook trout growth or MeHg concentration were larger than means for Atlantic salmon. The solid line is the linear regression fit, the shaded area shows the 95% confidence region for the mean predicted difference, and the dashed line shows no difference between the means for brook trout and Atlantic salmon.

Mean final mass of both species ranged widely across sites (brook trout: 1.9–8.5 g; Atlantic salmon: 1.6–6.9 g). Brook trout mean mass at the end of the growing season was not significantly affected by variation in the biomass of aquatic prey (Table 1), consistent with an energetic benefit of terrestrial resource subsidies. In contrast, Atlantic salmon mean mass was substantially suppressed at sites where aquatic prey biomass was low (Table 1). Both brook trout and Atlantic salmon mass were suppressed at high population density of conspecifics (Table 1). However, neither species’ mass was significantly suppressed at high population density of the other species (Table 1). The difference in mass between the species was related to prey biomass, ([mean log_10_ brook trout mass – mean log_10_ Atlantic salmon mass] = 0.20−0.22(log_10_ mg prey per sample), *r* ^2^ = 0.31, RMSE = 0.16, *F*_1,18_ = 8.0, *P* = 0.01) with brook trout mass highest relative to salmon at sites with low aquatic prey biomass (Figure 2).

**Table 1:** Multiple linear regression results for the relationship of young-of-the-year brook trout or Atlantic salmon growth (as mean log_10_ individual mass in fall) on population density and prey biomass (full model fit statistics: brook trout r^2^ = 0.68, F_3,16_ = 11.3, P = 0.0003; Atlantic salmon r^2^ = 0.71, F_3,16_ = 13.2, P = 0.0001).

Effect	Brook trout estimate (SE)	P	Atlantic salmon estimate (SE)	P
Intercept	0.97 (0.10)	<0.0001	0.69 (0.12)	<0.0001
log_10_(brook trout per 100 m^2^)	−0.53 (0.12)	0.0004	−0.08 (0.13)	0.55
log_10_(Atlantic salmon per 100 m^2^)	−0.01 (0.10)	0.89	−0.27 (0.12)	0.03
log_10_(prey mg per sample)	−0.01 (0.07)	0.91	0.34 (0.07)	0.0003

**Figure 3:**
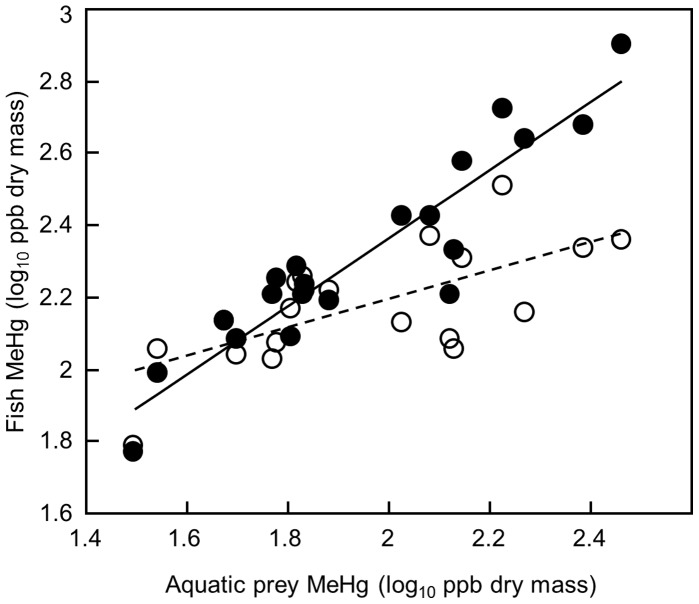
Brook trout are less contaminated than Atlantic salmon at sites with elevated contamination in prey. Points on the scatterplot show the average methylmercury (MeHg) concentrations in brook trout (open circles) and Atlantic salmon (closed circles) against MeHg concentration in aquatic prey (same prey value for both species at each site). Error bars are omitted for clarity; SEs of all estimates were less than 0.1 log units. Lines show the linear regression fits for brook trout (dashed line) and Atlantic salmon (solid line).

Terrestrial prey MeHg (mean: 17 ppb dry, SE: 4) was lower than aquatic prey MeHg (mean 106 ppb dry, SE 16; t-test including only sites with both aquatic and terrestrial prey samples *t* = −4.5, *P* = 0.01). Average aquatic prey MeHg varied widely across sites (range: 31–288 ppb dry), and was highest at sites with low prey biomass (log_10_ prey MeHg ppb dry = 2.2−0.29 (log_10_ mg prey per sample), *r* ^2^ = 0.26, RMSE = 0.24, *F*_1,18_ = 6.5, *P* = 0.02). Mean MeHg concentrations in brook trout varied less across sites than those in Atlantic salmon (brook trout: 61–330 ppb dry MeHg; Atlantic salmon: 60–820 ppb dry). MeHg concentrations in both brook trout and Atlantic salmon were correlated with MeHg concentrations in aquatic prey (brook trout: log_10_ brook trout MeHg ppb dry = 1.4+0.39 (log_10_ prey MeHg ppb dry), *r* ^2^ = 0.44, RMSE = 0.12, *F*_1,18_ = 14.7, *P* = 0.001; Atlantic salmon: log_10_ Atlantic salmon MeHg ppb dry = 0.48+0.94 (log_10_ prey MeHg ppb dry), *r* ^2^ = 0.86, RMSE = 0.10, *F*_1,18_ = 113.2, *P<*0.0001), with the highest concentrations in both species at unproductive sites where MeHg concentrations in aquatic prey were highest (Figure 3). However, while brook trout and Atlantic salmon MeHg concentrations were similar at productive sites with high aquatic prey biomass, brook trout MeHg concentrations were always lower than those in Atlantic salmon at low prey biomass sites where MeHg concentrations in prey were elevated (Figure 2; [mean log_10_ brook trout MeHg ppb dry – mean log_10_ Atlantic salmon MeHg ppb dry] = −0.34+0.27 (log_10_ mg prey per sample), *r* ^2^ = 0.49, RMSE = 0.13, *F*_1,18_ = 17.3, *P* = 0.0006). Lower MeHg concentrations in brook trout at these lower-productivity sites are consistent with a disproportionate shift to using low-MeHg terrestrial prey.

## Discussion

Food webs are not constrained by the boundaries between habitats [37]. For consumers in streams and nearby terrestrial areas, the reciprocal exchange of subsidies can support a substantial amount of secondary production [1] and provide a substantial load of relatively contaminated [3] or uncontaminated (this study, [6]) resources. Yet, the importance of subsidies clearly varies over space and time and among species [38], [39]. The next step in subsidy ecology is identifying the situations in which subsidies are likely to be particularly important for ecosystem processes, community structure, or the performance of particular species [7] and thus likely to be important for contaminant dynamics. We found that differential use of terrestrial resource subsidies by two fish species was associated with distinctive growth and contaminant accumulation responses of each species to a large-scale productivity gradient. Brook trout used more terrestrial resources than Atlantic salmon and were buffered against the effects of low in-stream productivity. These results have important implications for understanding the basis of stream fish production and ecological interactions between brook trout and Atlantic salmon, and for identifying sites and species likely to have elevated MeHg concentrations in fish. Further, this study shows that combined analyses of growth or biomass, stable isotopes, and contaminant tracers like MeHg are a promising technique for gaining detailed information about variation in the importance of subsidies and other food web relationships across species and sites (see also [40], [41], [42] for examples from other systems).

### Ecological Implications

Our results highlight two important determinants of the ecological role of terrestrial resource subsidies in stream ecosystems. First, use of terrestrial subsidies differed substantially between similar, co-occurring fishes. Brook trout used terrestrial resources more than salmon, and only brook trout were able to translate this subsidy into sustained growth rates at sites with low aquatic prey biomass. Other studies have reported that, where they coexist, brook trout use terrestrial resources to a greater degree than Atlantic salmon [9], [22], but no previous studies have extended this to evaluate the relative performance of the species across an in-stream productivity gradient. This pattern of diet differences could be driven by species-specific prey preference and foraging behavior or by competition, wherein brook trout exclude Atlantic salmon from terrestrial resource use (but see [22]) as has been observed for other salmonids [10]. In either case, direct benefits of terrestrial subsidies are limited to a subset of species with the foraging flexibility or competitive ability to utilize them. Similar patterns occur for the reciprocal subsidy, where particular taxa of terrestrial predators benefit disproportionately from emerging aquatic insects [43]. In general, the community and ecosystem-level effects of subsidies are likely to be contingent upon the responses of particular species that are suited to take advantage of them.

Second, the relative benefit for a species of taking advantage of terrestrial subsidies depended on productivity of the recipient habitat. Young-of-the-year brook trout tended to be smaller than Atlantic salmon at productive sites with high aquatic prey biomass, but averaged nearly three times the mass of Atlantic salmon at unproductive sites where Atlantic salmon growth was suppressed. There is no evidence for a strong general relationship between the importance of subsidies and the relative productivity in donor and recipient habitats in other studies [7]. However, a strong effect of recipient habitat productivity on the relative performance of species on the same trophic level is likely when the terrestrial subsidy and in-stream resources are of similar types (e.g. drifting insects).

Subsidies may also play an important role in determining the outcome of population-level interactions. Growth of both brook trout and Atlantic salmon was suppressed at high conspecific population density, suggesting that there is intraspecific competition for juvenile growth resources (i.e. prey or foraging habitat [44]). However, there was no evidence of interspecific competition–high population density of one species did not have a significant effect on growth of the other. Our results, along with other studies that examine foraging in detail [9], indicate that diet segregation, particularly when aquatic prey is rare, plays a role in alleviating potential competition between young brook trout and Atlantic salmon. Further, the context-dependent differences between the growth of YOY brook trout and Atlantic salmon across a productivity gradient may represent an important competitive trade-off for older life stages. The outcome of competition between salmonid species is often driven by size asymmetry [45], [46]. Due to the differential response to variation in in-stream productivity, neither brook trout nor Atlantic salmon maintained a consistent juvenile size advantage across the productivity gradient, potentially promoting coexistence in stream environments well-known for spatial variation in aquatic prey biomass and terrestrial subsidy supply [2], [30].

### Bioaccumulation Implications

Concentrations of MeHg in fish are determined by MeHg concentrations in the food consumed and the trophic transfer rate. Based on the isotope-estimated differences in diet between brook trout and Atlantic salmon and the observed lower MeHg concentrations in terrestrially-derived prey, we conclude that differences in MeHg in food led to lower concentrations of MeHg in brook trout at low-productivity sites. But could trophic transfer rates to brook trout also have been lower than for Atlantic salmon? The two species had similar MeHg concentrations at sites with high aquatic prey biomass, where brook trout and Atlantic salmon diets were likely similar, suggesting that trophic transfer to brook trout is not inherently lower than salmon. However, trophic transfer can be reduced by rapid, efficient growth via growth dilution [17] and brook trout did generally grow larger than salmon at low-productivity sites. Further, brook trout may have lower activity costs of metabolism and higher growth efficiency than Atlantic salmon [47]. Thus, growth dilution may have contributed to lower MeHg concentrations in brook trout than Atlantic salmon at resource-poor sites. Such effects would be further magnified if high MeHg in Atlantic salmon led to reduced growth efficiency, but the MeHg concentrations we observed are not within the range known to affect fish performance [16].

Aquatic ecosystems are hotspots for accumulation of MeHg and other contaminants, and the potential risk posed by the export of these aquatic contaminants to terrestrial organisms (including humans) is an ongoing concern. We show that the reciprocal pathway of terrestrial inputs into aquatic ecosystems may play a key role in driving patterns of contaminant accumulation by diluting contaminant uptake in some aquatic consumers (see also [6]). Similar resource flux between food webs with low and high contaminant concentrations occur in other aquatic systems, such as between the benthic and pelagic zones of lakes and estuaries [41], [48]. As we saw in this study, the importance of subsidies between different food webs for contaminant dynamics in these other systems is likely to vary depending on their relative productivity and may be different for sympatric species with different foraging behavior. Therefore, in order to accurately predict the risk of contaminant exposure, we will need to move beyond studies of the physical and chemical drivers of contaminant input and availability and understand how subsidies influence the food web pathways of contaminant accumulation and dilution.
